# Current saturation in free-air ionization chambers with chopped synchrotron radiation

**DOI:** 10.1107/S0909049513016154

**Published:** 2013-07-03

**Authors:** Nobuteru Nariyama

**Affiliations:** a Light Source and Optics Division, Japan Synchrotron Radiation Research Institute, Kouto 1-1-1, Sayo, Hyogo 679-5198, Japan

**Keywords:** ionization chamber, X-ray chopper, ion recombination, pulsed X-rays

## Abstract

Saturation curves for free-air ionization chambers with electrode gap widths of 4.2, 8.4 and 18 mm were obtained for 10 and 15 keV undulator synchrotron radiation thinned with a 230 Hz rotating-disk chopper. Ion recombination in free-air ionization chambers was found to be inversely proportional to the applied electric field, and an expression that satisfactorily reproduced the ion-recombination rate is determined. A comparison of the expressions for continuous and pulsed X-rays revealed that chopped high-intensity X-rays require a higher voltage to attain saturation when the product of the pulse width and electric field exceeds a value that depends on the X-ray energy. This behaviour was observed explicitly for 10 keV X-rays in measurements with the ionization chamber placed before and after the chopper.

## Abstract 2

An expression for ion recombination in free-air ionization chambers irradiated by chopped X-rays is presented. The expression is validated by comparison with experiments using synchrotron radiation.

## 1. Introduction   

At synchrotron radiation facilities, a chopper is used to thin out X-rays and to alter the time structure for particular time-resolved experiments. As a result, the X-ray dose rate is inevitably reduced as the X-rays are thinned, so the necessary voltage to prevent ion recombination in a free-air ionization chamber monitoring current pulses is expected to be lower. However, owing to the modified time structure, the predicted performance cannot necessarily be guaranteed.

Ordinarily, an ionization chamber is operated in current mode. The chamber has its own time response corresponding to the ion transit time, which depends on the width of the electrode gap and applied voltage. The ion transit time is generally much longer than the several tens to several hundred nanosecond interval between electron bunches in the storage rings. However, thinning of the X-rays makes the interval between the X-ray pulses longer (2–20 ms), while the pulse duration, *i.e.* the time for which the chopper windows are open, is kept shorter than the ion transit time of the chamber. Under these conditions, X-rays behave as pulsed X-rays in the ionization chamber. For pulsed X-rays, ion loss is known to be linearly proportional to 1/*V*, where *V* is the applied voltage. In contrast, for continuous X-rays or synchrotron radiation from a storage ring, ion loss is proportional to 1/*V* ^2^ (Boag, 1966 ▶). This weak dependence on the voltage suggests that, as the X-ray intensity increased, current saturation, at which point negligible ion recombination occurs owing to the high voltage being applied, becomes more difficult to achieve for pulsed X-rays than for continuous X-rays. Therefore, contrary to general expectations, a chopper increases the voltage necessary for intense X-rays, regardless of the large reduction in dose rate.

We previously measured the collection efficiency in free-air ionization chambers for synchrotron X-rays chopped using a rotating disk with narrow slits. Then, by applying the expression for a parallel-plate ionization chamber, we found that the ion-recombination rate was proportional to the X-ray intensity and inversely proportional to the applied voltage (Nariyama, 2012 ▶). In that work, however, the dependence of the ion-recombination expression on the electrode spacing was not fully explored. In the model, broad X-rays are incident on the electrodes such that ions are produced uniformly throughout the gap. In free-air ionization chambers, through which gas is often circulated at beamlines, the narrow X-ray beam travels between two electrodes set parallel to the beam; ions are produced only in part of the gap and this limited volume must be considered. Thus, this difference between parallel-plate and free-air ionization chambers requires that the ion recombination expression used in our previous study be revised accordingly for free-air ionization chambers.

In this paper, an expression for the ion-recombination correction for a free-air ionization chamber with a pulsed photon beam is proposed by modifying Boag’s expression, and its validity is investigated by comparison with experiments using chambers with different gap widths and thinning synchrotron radiation beams. Current-saturation conditions for chopped high-intensity X-rays are also discussed.

## 2. Materials and methods   

### 2.1. Derivation of the ion recombination expression for free-air ionization chambers with pulsed X-rays   

**Figure 1:**
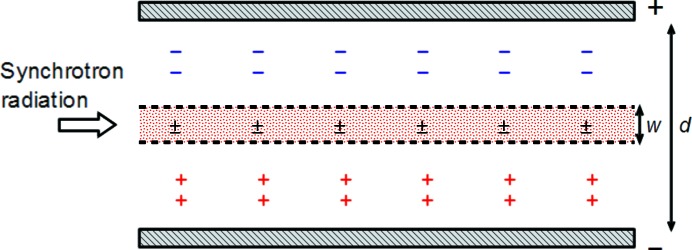
Ion distribution in a free-air ionization chamber exposed to a pulsed photon beam. The regions above and below *w* contain only negative and positive ions, respectively. Recombination occurs only in the overlapping region *w*, which shrinks with time from its initial value of *w*_0_.

Fig. 1 ▶ shows a schematic of the positive and negative ions present in a free-air ionization chamber during charge collection after the passage of a pulsed beam, where the pulse duration is assumed to be much shorter and the pulse interval longer than the ion transit time across the chamber. While the distribution is similar to that of a parallel-plate chamber (Boag, 1966 ▶), only part of the sensitive region is irradiated directly with X-rays in the free-air ionization chamber. This difference thus necessitates the introduction of a parameter reflecting the size of the irradiated region.

Recombination occurs only in a limited region where both positive and negative ions are present, as indicated by the dotted area in Fig. 1 ▶, and the recombination rate can be expressed as
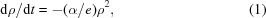
where ρ is the positive or negative ion density, α is the recombination coefficient and *e* is the electron charge. The density is then
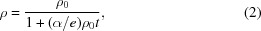
where ρ_0_ is the initial ion density per pulse. The overlapping region contracts at a constant rate over time; *i.e.* the width *w* decreases as
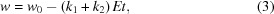
where *w*_0_ is the initial width of the overlapping region, *k*_1_ and *k*_2_ denote positive and negative ion mobilities, and *E* is the applied electric field. The quantity *w*_0_ is equal to the separation distance *d* in a parallel-plate chamber because ions are produced over the whole of *d*. In the free-air ionization chamber, however, the width is restricted by the shorter of the two distances, *d* or the produced electron range. The time at which the overlap region disappears (*i.e.* *w* = 0) is
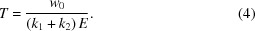
Therefore, the number of recombinations is given by
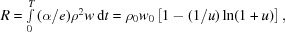
where
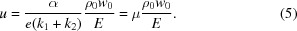
For a parallel-plate ionization chamber, *u* is equal to μρ_0_ *d*/*E*. Hence the collection efficiency *f* is
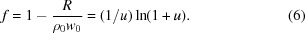
Near saturation, the parameter *u* is less than 0.1, and all terms of order *u* ^2^ and higher can be neglected in the series expansion. As a result, equation (6) can be approximated as
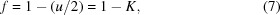
where *K* represents a factor to correct for ion recombination.

### 2.2. Comparison with the expression for continuous X-rays   

Before chopping, synchrotron radiation from a storage ring is considered to be continuous. In this case, the expression for ion recombination is given by (Scott & Greening, 1961 ▶; Niatel, 1967 ▶)
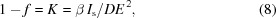
with
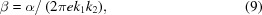
where *I*_s_ is the saturation current and *D* is the electrode length. The parameter β is constant at low electric field strength but decreases as the electric field strength is increased (Nariyama, 2006 ▶), in the same way as μ; this is attributable to the increasing fraction of free electrons that reach the electrode without attaching to oxygen molecules (ICRU, 1982 ▶).

While at first glance equation (8) appears to be different from Boag’s equation for a parallel-plate ionization chamber (Boag, 1966 ▶), it should be noted that *I*_s_/*D* is proportional to *a* ^2^ *q*, where *a* is the spatial size of the ionized region and *q* is the ion charge liberated per unit volume per second. Thus, for a narrow beam, *K* is proportional to *a* ^2^ *q*/*E* ^2^, and equation (8) is then equivalent to Boag’s equation for continuous X-rays.

Here, we assume that ion recombination, *K*, in a free-air ionization chamber using pulsed X-rays produced by chopping synchrotron radiation exceeds that of using the original synchrotron radiation, even at the same applied voltages and under the condition that *K* is below 2%; *i.e.* the ratio of *K* in equations (7) and (8) is greater than unity. As *I*_s_ is equal to ρ_0_ integrated over the ionizing volume and divided by the pulse duration, *t*_w_, the following relation can be deduced,
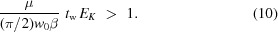
In this expression, *E*_*K*_, which is the electric field that keeps the value of *K* low enough, must increase with the X-ray intensity, demonstrating that chopping X-rays increases ion recombination at high X-ray intensities.

### 2.3. Saturation-curve experiments   

To verify expression (7), experimental data obtained at the undulator beamline BL09XU at SPring-8 (Nariyama, 2012 ▶) were employed. The measurements considered two types of ionization chambers: IC1 (FA-105; Applied Engineering Inc.) and IC2 (FA-105a). However, as the difference between the spacings in the two chambers (4.2 and 8.4 mm) was not large enough to unambiguously distinguish the effects of spacing on ion recombination, measurements using a third ionization chamber, IC3 (S-1194C1; Ohyo Koken Kogyo Co.), with an 18 mm gap were also performed. The electrode length and width were 5 and 30 mm for IC1 and IC2, and 140 and 60 mm for IC3.

For the experiments with IC1 and IC2, the storage ring was operated in multi-bunch mode, in which twelve 160-bunch trains travel around the ring at 85 ns intervals. As the multi-bunch mode was not available for the experiments with IC3, the several-bunch A mode was used, in which 203 bunches circled at intervals of 23.6 ns. Monoenergetic photons of 10 and 15 keV were produced from the synchrotron radiation through a double-crystal monochromator, and the intensity without chopping was 4 × 10^12^–8 × 10^12^ photons s^−1^ at 10 keV and 4 × 10^12^ photons s^−1^ at 15 keV.

To chop the beam, the same rotating disk was used in each case; the chopper was a 1 mm-thick stainless steel disk with two 0.1 mm-wide slits and two 0.36 mm-wide slits positioned at radii of 55 and 50 mm, respectively. The 0.1 mm-wide slits were used in all experiments except those discussed in §3.3. The rotating speed was controlled by the voltage applied to the DC motor (MM-10CN, Nippo Denki Co.); a typical speed of 6900 r.p.m. produced a beam with a duration of 2.5 µs at 230 Hz, *i.e.* a 4.3 ms interval. The slit size determined the height of the beam, and the width was set to 0.5 mm using the beamline XY slit.

**Figure 2:**
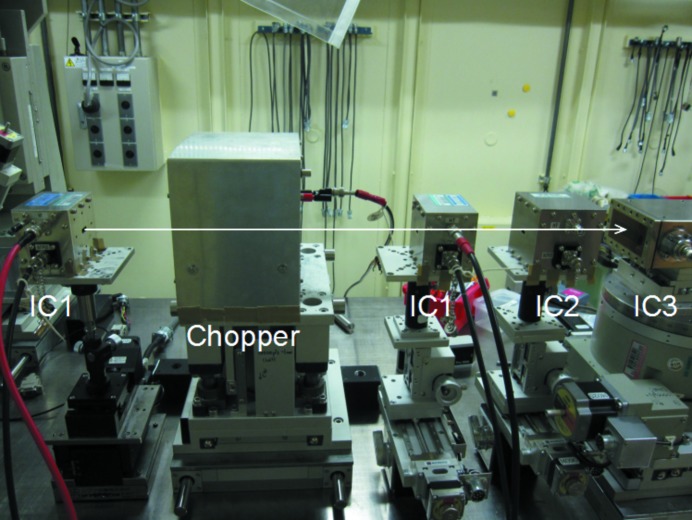
Experimental set-up. The beam comes from the left side, through IC1, the rotating chopper, IC1, IC2 and IC3 in that order.

The various free-air ionization chambers were used in parallel as shown in Fig. 2 ▶. The incident X-ray intensity and intensity variation were monitored with IC1, which was located after the chopper. The ionizing current at negative applied voltage was measured each second with an electrometer (Keithley 6517A) and averaged over 1 min.

## 3. Results and discussion   

### 3.1. Confirmation of the chamber response to pulsed X-rays   

**Figure 3:**
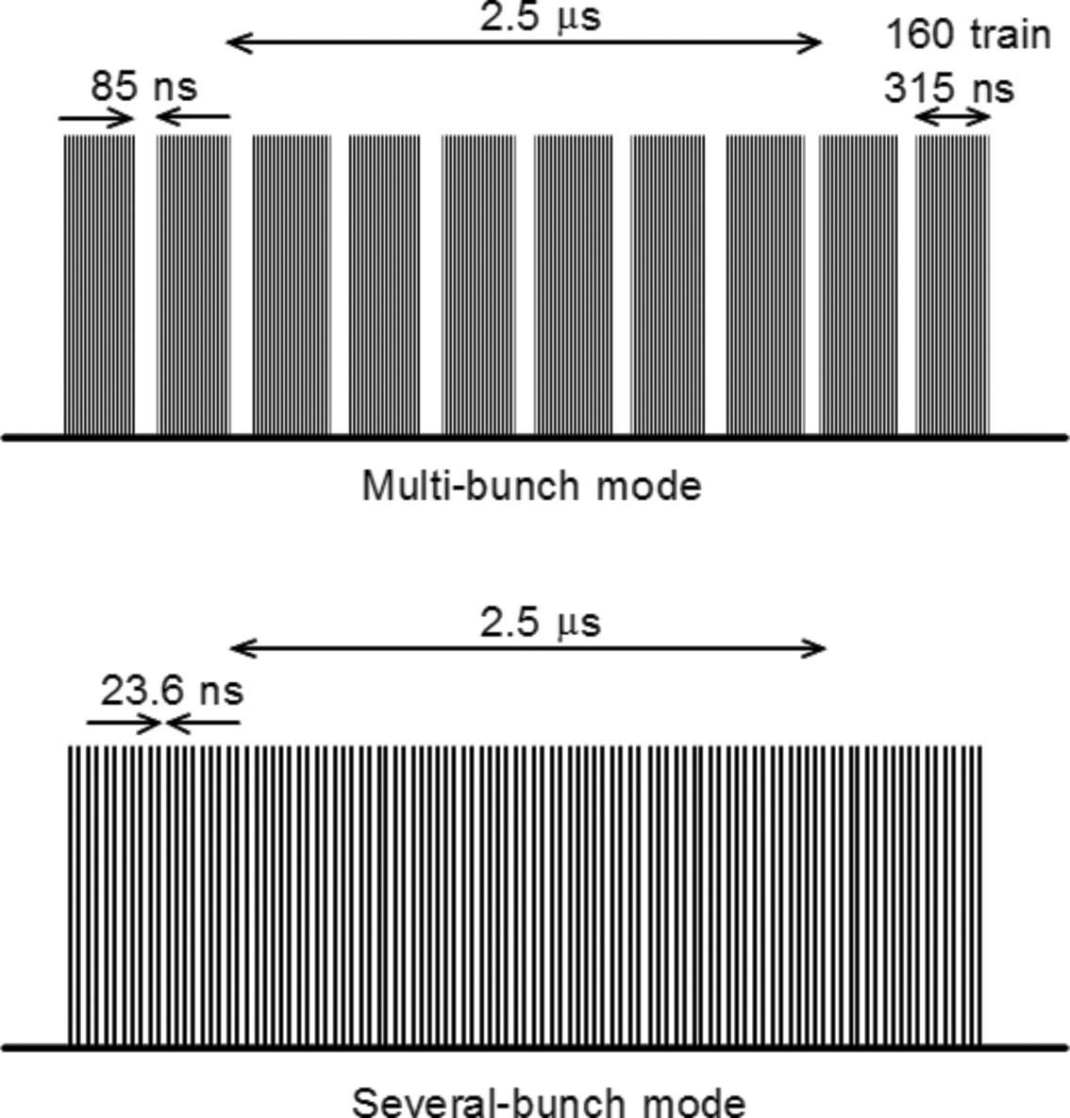
Scheme showing the relationship between bunch interval, duration and the shutter opening of 2.5 µs for both operating modes. In the multi-bunch mode, 315 ns-long bunches consisting of 160 trains come every 85 ns. In the several-bunch mode, single bunches come every 23.6 ns. As the ion-collection time was as long as 33 µs for IC1 at 3 kV, the X-rays coming through the 2.5 µs window can be considered as a single X-ray pulse.

In the multi-bunch and several-bunch modes, the X-rays from each bunch were incident every 85 and 23.6 ns, respectively; that is, opening the slit for 2.5 µs resulted in 6.25 and 106 X-ray ‘flashes’, as shown schematically in Fig. 3 ▶. The pulse duration was about 50 ps for the single bunch, and for the multi-bunches the interval between bunches in the bunch train was 2.0 ns. The intervals and duration were much shorter than the ion-collection time; the shortest collection times were 33, 132 and 608 µs for IC1, IC2 and IC3 at −3 kV (ICRU, 1982 ▶). X-rays are considered to be pulsed when the pulse width is shorter and the interval between pulses is longer than the ion transit time across the chamber. By this definition, the X-rays over adjacent bunches were continuous and the intermittent incident X-rays during the 2.5 µs opening were pulsed.

**Figure 4:**
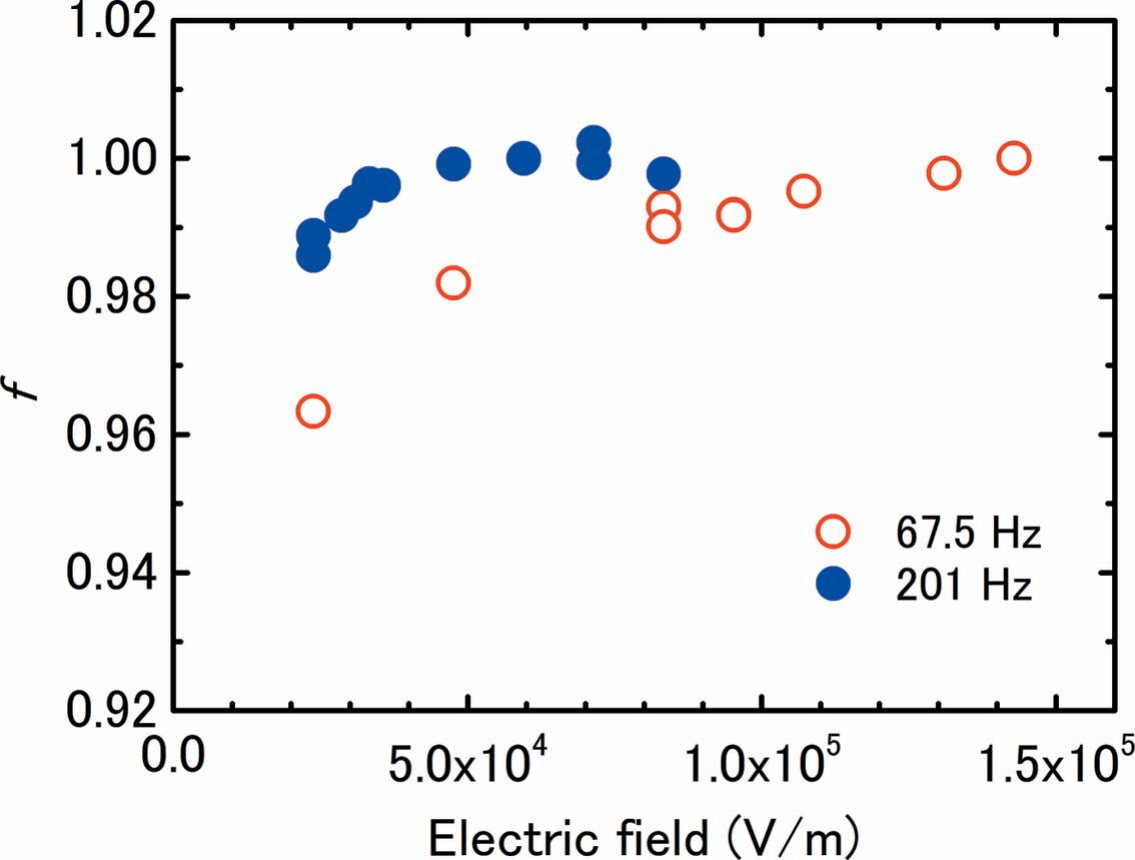
Current-saturation curves for IC1 at different rotation speeds for 15 keV X-rays. The pulse repetition frequencies were 67.5 and 201 Hz. The charges per pulse were 1.73 and 0.622 pC, whereas the currents were almost the same.

To confirm that the response of the chambers to the chopped X-rays was the same as that to an X-ray pulse, saturation curves were measured at different rotating speeds for the same beam. Under these conditions the charge per second (*i.e.* the current) did not vary as the charge per pulse was increased by decreasing the rotating speed. If the response of the chamber is the same as that for continuous X-rays, then the same saturation curves will be obtained for any rotating speed. Fig. 4 ▶ shows the saturation curves for pulse repetition frequencies of 67.5 and 201 Hz with 15 keV X-rays. At the faster rotation speed, the current was saturated at 0.25 kV, whereas saturation was not attained even at 0.6 kV and the slower rotation speed, demonstrating that the response of the chambers corresponds to X-ray pulses and not continuous X-rays.

### 3.2. Comparison of the experiment with the derived expression   

**Figure 5:**
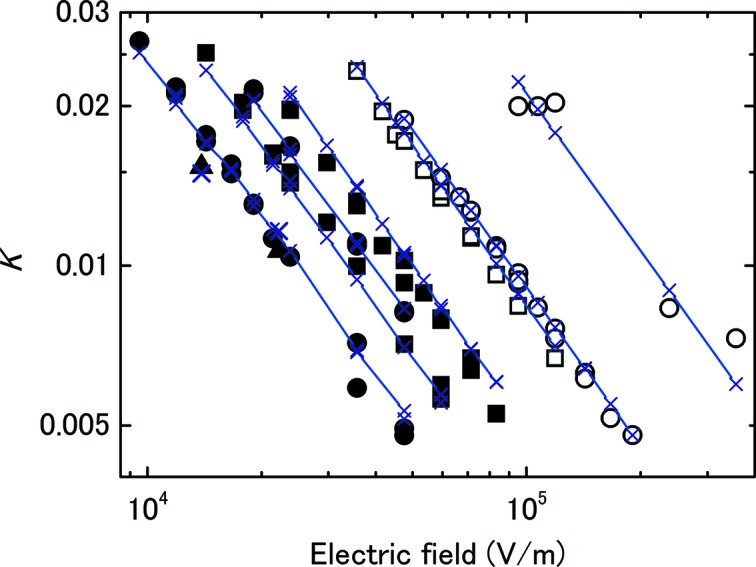
Plots of *K* against *E* demonstrating that *K* is inversely proportional to *E*. The circles, squares and triangles denote IC1, IC2 and IC3, respectively. The open and closed marks are data for 10 and 15 keV X-rays, respectively. The lines with crosses denote the calculated *K* values using equation (7), in which the value of 3.02 × 10^10^ V m C^−1^ was used for μ (ICRU, 1982 ▶). The charge per pulse ranged from 0.4 to 1.7 pC.

Equation (7) can be rewritten as
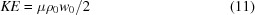
to highlight that, if (7) holds, *K* is inversely proportional to *E*. Fig. 5 ▶ shows a plot of *K* and *E* for the three ionization chambers with photon intensities of 10 and 15 keV obtained for measurements with *K* varied between 0.5% and 2%. The charge per pulse ranged from 0.4 to 1.7 pC. The inverse proportionality of *K* and *E* is clearly observed.

To calculate the values of *K* using equation (7), it is necessary to know the initial ion distribution area in order to define *ρ*_0_ and *w*_0_; this depends on the photon energy, *i.e.* the maximum range within which electrons are ionized. When the electrode gap is narrower than this range, the area is limited by the electrodes. The energy deposition distribution was calculated using the Monte Carlo code *PENELOPE2011* (Sempau *et al.*, 1997 ▶) assuming that 10 and 15 keV square beams with a side of 0.1 mm were incident on the air between the iron electrodes. Photon and electron movements were traced down to 1 and 0.1 keV, respectively. The result showed that 99% of the total energy deposited was absorbed within a 3.0 mm-diameter area of air at 10 keV and a 6.36 mm-diameter area at 15 keV. Thus, these values defined the initial charge area: a 3.0 mm-diameter area at 10 keV and a 6.36 mm-diameter area at 15 keV for IC2 and IC3, and a 4.2 mm (vertical) by 6.36 mm (horizontal) area for IC1 at 15 keV.

For ρ_0_, instant generation of a uniform charge density is assumed (ICRU, 1982 ▶). For the pulse duration of 2.5 µs, however, the width of the overlapping region *w* shrinks by up to 0.65 mm. Owing to the time lag, the initial charge density does not precisely correspond to the charge liberated in the time during which the window is open and is weakened somewhat. In previous investigations of the recombination correction for pulsed beams, similar conditions have been used; in the studies by Martino *et al.* (2005 ▶) and Bruggmoser *et al.* (2007 ▶), the pulse length was 4 µs for ion-collection times of 90–260 µs and 20–170 µs, respectively. In those studies, the time lag effect was neglected and the charge was obtained by dividing the dose integrated over a defined time interval by the number of accelerator pulses. This method to calculate the charge was also adopted here.

Using these values for ρ_0_ and *w*_0_, values of *K* were calculated from the measured data. Fig. 5 ▶ shows the results. For the μ value, 3.02 × 10^10^ V m C^−1^ has been recommended for experiments at 1 atm with an electrode spacing of no less than 2.5 mm (ICRU, 1982 ▶). The deviation in the line on the left was the result of fluctuations in the photon intensity because the measured data were used for the charge per pulse. The calculated value of *K* agreed with the measured value to within ±50%.

### 3.3. Comparison of saturation curves for high-intensity X-rays with and without the chopper   

**Figure 6:**
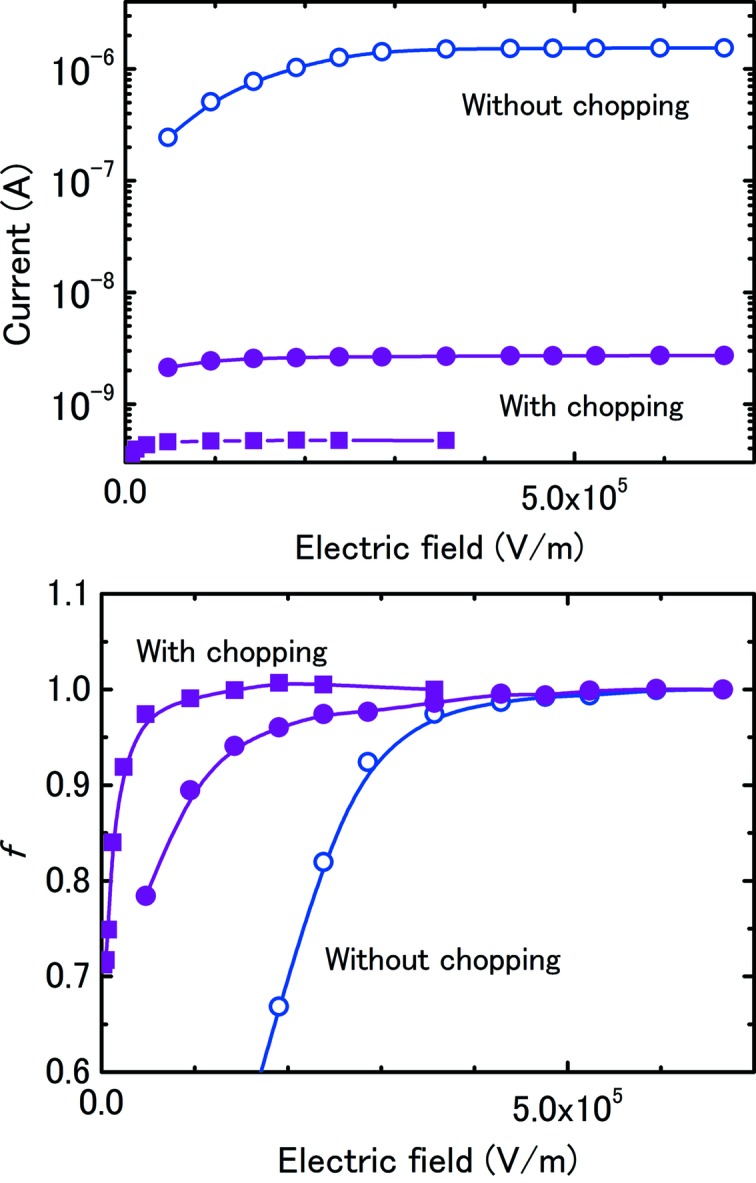
Current-saturation curves for 10 keV X-rays in two IC1 chambers placed before and after the chopper. The rotating speed was 181 Hz and the slit size was 0.36 mm; as a result, the window-opening period was 11.5 µs. The low-current data with chopping were obtained through the 0.1 mm slit. Both the current (top) and collection efficiency *f* (bottom) are shown as a function of the applied electric field.

For 10 keV X-rays incident on an ionization chamber with a 4.2 mm gap, equation (10) reduces to the following simple relation, where 5 × 10^13^ V^2^ A^−1^ m^−1^ has been substituted for β (Niatel, 1967 ▶; Nariyama, 2006 ▶) and 3 × 10^10^ V m C^−1^ for μ (ICRU, 1982 ▶),
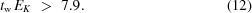
Fig. 6 ▶ shows the saturation curves during the 10 keV X-ray irradiation of two IC1 chambers placed before and after the chopper. The 0.36 mm slit was used to increase the charge per pulse with *t*_w_ equal to 11.5 µs. The photon intensity before the chopper was 4.0 × 10^13^ photons s^−1^ and the charge per pulse after the chopper was 15 pC. While the measured saturation current without chopping was 571 times larger than that with chopping as shown in Fig. 6 ▶ (top), the saturated electric field was almost the same at about 6.7 × 10^5^ V m^−1^, as shown in Fig. 6 ▶ (bottom). The curve for continuous X-rays rises steeply in contrast with the slow increase for pulsed X-rays due to the respective 1/*V* ^2^ and 1/*V* behaviours. From the measured data, the currents before the chopper at electric field strengths of 5.2 × 10^5^ V m^−1^ and 6.0 × 10^5^ V m^−1^ were only 0.6% and 0.05% smaller, respectively, than that at 6.7 × 10^5^ V m^−1^. In the figure, a curve for chopped X-rays produced using the smaller 0.1 mm slit is also shown. *E*_*K*_ was clearly lower than that for unchopped X-rays owing to the low ionizing current. Thus, the behaviour expected from equation (12) was confirmed experimentally, although the *t*_w_ *E*_*K*_ value of 7.7 s V m^−1^ was slightly smaller than predicted by (12).

### 3.4. Dependence of *E*_*K*_ on the unchopped X-ray intensity and pulse width   

**Figure 7:**
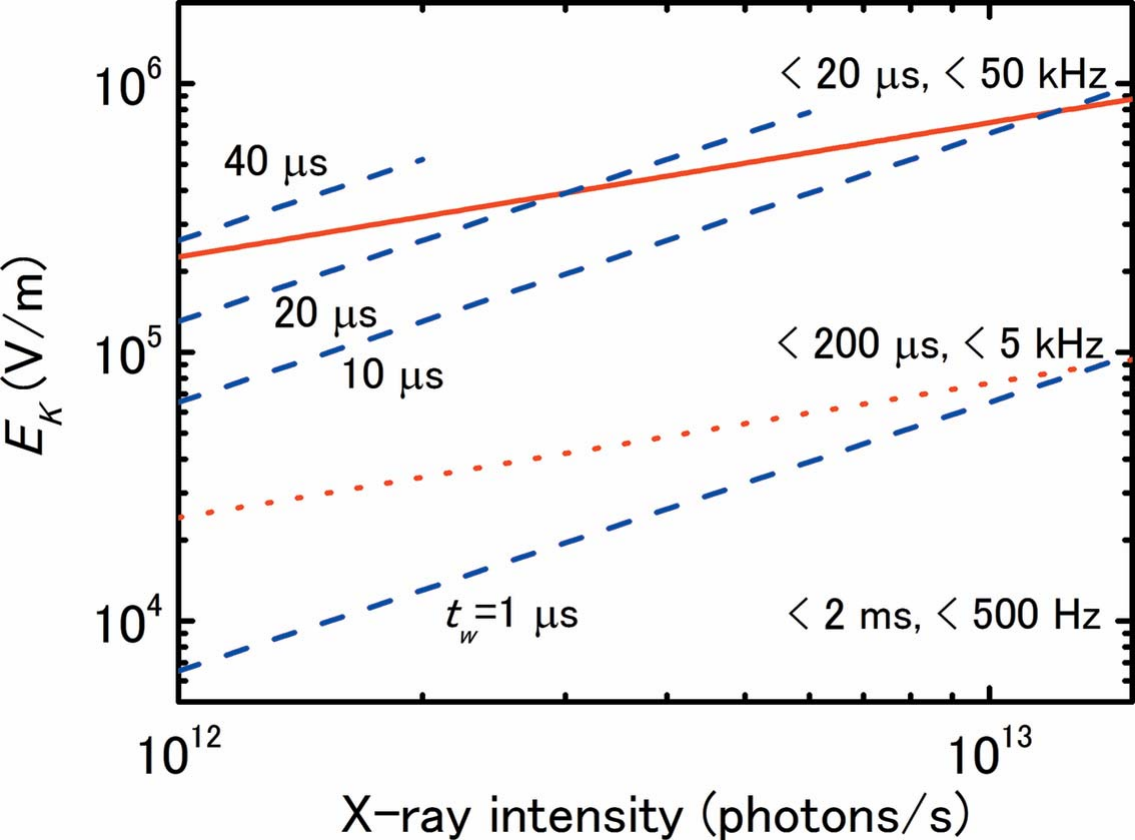
Schematic plot of *E*_*K*_ at *d* = 4.2 mm for *K* = 0.005 with and without chopping as a function of the unchopped X-ray intensity at 10 keV. The solid and dashed lines express the equation of the continuous and pulsed X-rays, respectively. The pulse duration, *t*_w_, was varied from 1 to 40 µs. The dotted line expresses the calculated value of *E*_*K*_ assuming that the X-rays at *t*_w_ = 10 µs are continuous. The time and frequency on the right-hand side indicate the ion-collection time for that *E*_*K*_ value and the corresponding frequency.

Fig. 7 ▶ schematically shows the calculated value of *E*_*K*_ for current saturation at *d* = 4.2 mm with and without chopping as a function of the 10 keV X-ray intensity, where equations (7) and (8) were used assuming *K* = 0.005 and varying the pulse duration *t*_w_ from 1 to 40 µs. In the figure, with increasing *t*_w_, *E*_*K*_ also increases and exceeds the value without chopping at the crossing point of the solid and dashed lines; this condition satisfies equation (12). The value of *E*_*K*_ at the crossing point increases as *t*_w_ decreases. For example, when *t*_w_ is 10 µs, the value of *E*_*K*_ that satisfies equation (12) is 7.9 × 10^5^ V m^−1^, and the intensity at this electric field when *K* is 0.005 is 1.2 × 10^13^ photons s^−1^. When *t*_w_ is 1 µs, however, equation (12) does not hold unless *E*_*K*_ is 7.9 × 10^6^ V m^−1^, *i.e.* 33 kV, at 1.2 × 10^14^ photons s^−1^, which is too high for practical applications.

Here, it should be noted that, as *E*_*K*_ increases, the ion-collection time is simultaneously reduced. The time for IC1 (Nariyama, 2012 ▶) is indicated in the figure. When the pulse duration is comparable with or longer than the ion transit time across the chamber, the treatment becomes more complex (Boag, 1987 ▶). When the pulse duration becomes much longer than the ion transit time, however, the X-rays can be treated as continuous regardless of chopping; the current without chopping is used for *I*_s_ in equation (8). For this reason the dashed lines for *t*_w_ = 20 and 40 µs end at the electric field at which the pulse duration is of the same order as the ion transit time. Similarly, weak X-rays also act as continuous beams regardless of chopping because the slow ion-collection time at the low saturation voltage exceeds the interval between pulses. These considerations are, however, only theoretical; the long pulse duration or chopping of weak X-rays would not be used in most applications.

Here, it could be assumed that chopped X-rays with *t*_w_ = 10 µs can be considered continuous and the intensity, *I*_s_, is simply weak in proportion to the slit opening period; however, the calculated *E*_*K*_ is much lower than the measured value, as shown in Fig. 7 ▶. This is further evidence that chopped X-rays behave as pulsed X-rays.

### 3.5. Effects of space charge and free electrons   

The space-charge effect, in which the ionic space charge formed in the positive and negative layers weakens the effective electric field, has been considered by previous studies to be an important factor affecting ion recombination. In particular, it was believed that high-intensity X-rays produced a dense ion density, which possibly induces an intense space charge. The charge distribution near the electrode, however, is proportional to *qd*/*k*_1_ *E* or *qd*/*k*_2_ *E* for continuous X-rays (Scott & Greening, 1961 ▶), so that the magnitude of the space-charge effect depends on the recombination rate rather than the X-ray intensity. For continuous X-rays, Boag (1987 ▶) referred to the existence of a significant distortion in the electric field by space charge whenever there is a significant amount of recombination (more than 5%). In our measurements, recombination rates of less than 2% were used. For this small recombination rate (*i.e.* at high electric field), free electrons that do not attach to oxygen molecules play an important role, as has been shown *via* the pulse shape (Nariyama, 2012 ▶). Owing to this effect, a lower electric field than expected has been observed at saturation with high-intensity X-rays (Nariyama, 2006 ▶).

For pulsed X-rays, a positive charge cloud adjacent to the negative electrode reduces the field strength in the overlap region of the positive and negative charge distribution after the instantaneous clearance of free electrons. It has been concluded that this effect is insignificant in small ionization chambers at a dose level of 10 mGy per pulse (Boag *et al.*, 1996 ▶), which was the typical dose used in this work. While the effect remains important, recent investigations and our study have suggested that the free electrons play the primary role in the estimation of ion recombination at high electric fields.

**Figure 8:**
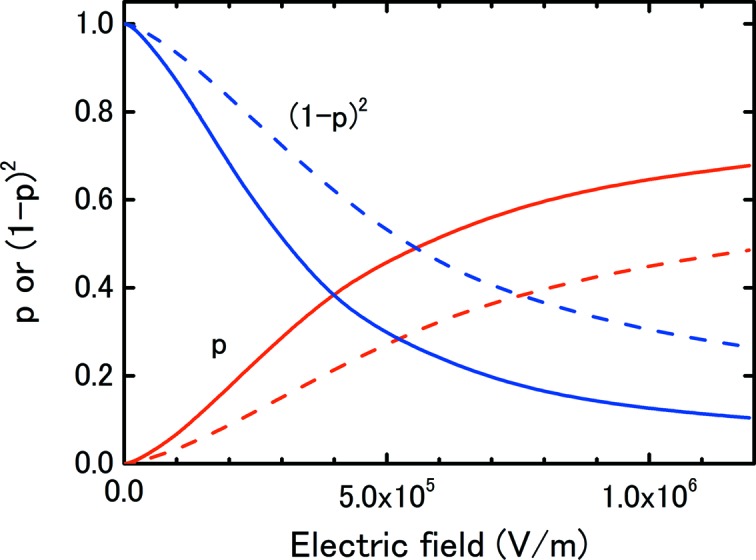
Free-electron fraction, *p*, and (1 − *p*)^2^ as a function of the electric field calculated using equations (14) and (15). The solid and broken lines indicate the results for *d* = 4.2 and 8.4 mm. The factor of (1 − *p*)^2^ is the ratio of *K* in equation (6) to that in equation (13), *i.e.* the rate of change of *K*.

For pulsed X-rays, three ways of expressing the collection efficiency corrected for unattached electrons have been proposed (Boag *et al.*, 1996 ▶). Each approach gave similar results, and here the following expression was used,
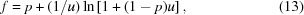
where *p* is the proportion of negative charge carriers that are free electrons. The parameter *p* can be calculated by (Boag & Wilson, 1952 ▶)
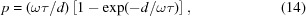
where ω and τ are the drift velocity and lifetime of the free electrons and depend on the electric field. From the calculated values of ω and measured values of τ in air, fitting expressions have been deduced (Hochhauser *et al.*, 1994 ▶; Laitano *et al.*, 2006 ▶). Fig. 8 ▶ shows the free-electron fraction at IC1 and IC2 calculated using this expression. Even at the same electric field, the free-electron fraction differs for the two gaps as explicitly shown in (13); the fraction becomes larger at the narrower gap because the free electrons arrive at the electrode sooner.

As with equation (7), equation (13) can be similarly approximated by
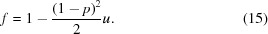
Compared with equation (7), *K* decreases by a factor of (1 − *p*)^2^. At an electric field strength of 4 × 10^5^ V m^−1^, the fraction of free electrons is about 40%, and (1 − *p*)^2^ is 0.4 at IC1, as shown in Fig. 8 ▶, meaning that *K* decreases by a factor of 0.4. This magnitude seemed to be larger than that observed in Fig. 5 ▶, but suggests that the effect was important.

## 4. Conclusions   

A modified expression corrected for ion recombination in free-air ionization chambers has been derived for a pulsed photon beam, and compared with experimental data using thinned synchrotron radiation. The collection efficiencies were measured at various pulse charges, electrode spacings and X-ray energies. Comparisons showed that the value of *K* agreed with the modified expression within reasonable limits even though there was some ambiguity in the initial ion distribution width and ion charge. A comparison with the expression for continuous X-rays revealed that, for high-intensity X-rays, a higher voltage is required for current saturation when chopping is applied than that without chopping. The results of this study demonstrate that such a situation could occur in spite of the significant decrease in the measured ionizing current. Therefore unfeasibly high voltages are necessary for the saturation of X-ray lasers that rely on extremely high-intensity pulsed X-rays (Nariyama, 2012 ▶).
